# Magnetic Susceptibility Source Separation Solely from Gradient Echo Data: Histological Validation

**DOI:** 10.3390/tomography8030127

**Published:** 2022-06-14

**Authors:** Alexey V. Dimov, Kelly M. Gillen, Thanh D. Nguyen, Jerry Kang, Ria Sharma, David Pitt, Susan A. Gauthier, Yi Wang

**Affiliations:** 1 Department of Radiology, Weill Cornell Medicine, New York, NY 10065, USA; ald2031@med.cornell.edu (A.V.D.); keg2002@med.cornell.edu (K.M.G.); tdn2001@med.cornell.edu (T.D.N.); jerry.kang@stonybrook.edu (J.K.); ria.sharma@danahall.org (R.S.); 2 Department of Neurology, Yale Medicine, New Haven, CT 06511, USA; david.pitt@yale.edu; 3 Department of Neurology, Weill Cornell Medicine, New York, NY 10022, USA; sag2015@med.cornell.edu; 4 Meinig School of Biomedical Engineering, Cornell University, Ithaca, NY 14850, USA

**Keywords:** quantitative susceptibility mapping, susceptibility source separation, myelin quantification, iron quantification

## Abstract

Quantitative susceptibility mapping (QSM) facilitates mapping of the bulk magnetic susceptibility of tissue from the phase of complex gradient echo (GRE) MRI data. QSM phase processing combined with an $R_{2}^{*}$ model of magnitude of multiecho gradient echo data ($R_{2}^{*}QSM$) allows separation of dia- and para-magnetic components (e.g., myelin and iron) that contribute constructively to$\;R_{2}^{*}$ value but destructively to the QSM value of a voxel. This $R_{2}^{*}QSM$ technique is validated against quantitative histology—optical density of myelin basic protein and Perls’ iron histological stains of rim and core of 10 ex vivo multiple sclerosis lesions, as well as neighboring normal appearing white matter. We found that $R_{2}^{*}QSM$ source maps are in good qualitative agreement with histology, e.g., showing increased iron concentration at the edge of the rim+ lesions and myelin loss in the lesions’ core. Furthermore, our results indicate statistically significant correlation between paramagnetic and diamagnetic tissue components estimated with $R_{2}^{*}QSM$ and optical densities of Perls’ and MPB stains. These findings provide direct support for the use of $R_{2}^{*}QSM$ magnetic source separation based solely on GRE complex data to characterize MS lesion composition.

## 1. Introduction

Multiple sclerosis (MS) is an autoimmune disease characterized by the presence of demyelinated lesions in the central nervous system. Progressing demyelination can be monitored using noninvasive MRI methods such as myelin water fraction mapping [1,2,3,4,5], and ongoing chronic inflammation can be detected by the presence of paramagnetic iron at the rims of MS lesions in susceptibility maps [6,7,8,9,10]. As both myelin loss and iron increase can colocalize within the lesion, interpretation of the apparent increase of magnetic susceptibility due to these processes is a non-trivial task [7,11]. Therefore, separate quantification of myelin and iron effects in MRI is essential for allowing specific in vivo monitoring of MS pathology.

Both myelin and iron can be quantified in MRI on the basis of their magnetic susceptibility. Quantitative susceptibility mapping (QSM) [12,13,14,15] maps distribution of the magnetic susceptibility of magnetic sources, such as iron, myelin, calcium, and exogenous contrast agents, requiring only the acquisition of complex gradient echo (GRE) data. As QSM measures the sum of susceptibilities of diamagnetic myelin and paramagnetic iron when they are present within the same voxel, separating the contributions of myelin with negative susceptibility and iron with positive susceptibility in QSM requires additional modeling and calibration of MR relaxation times [7,16,17,18,19,20]. However, these approaches require multiple calibrations and/or an additional spin echo $R_{1}$ or$\;R_{2}$ mapping, complicating workflow in practical applications. To address this shortcoming, recent work has been exploring the possibility of achieving quantification of positive and negative susceptibility sources based solely on GRE data using a three-sphere model but ignoring the necessary dipole-kernel-based spatial deconvolution in QSM [21].

Recently, we proposed an $R_{2}^{*}$-model of susceptibility from signal magnitude combined with QSM processing of phase ($R_{2}^{*}QSM$) that allows separation of susceptibility sources using only gradient echo data while preserving the dipole deconvolution in phase processing [22]. We demonstrated equivalence of the results obtained using our model for neural tissue in vivo with an $R_{2}^{\prime }$-model of susceptibility [19]. In this work, we further validate the $R_{2}^{*}QSM$ separation of magnetic sources by referencing it to quantitative histology.

## 2. Materials and Methods

### 2.1. MR Signal Modeling for $R_{2}^{*}QSM$

The complex signal exponent of the gradient echo MRI signal is approximated as [19,22,23]:

(1) $$R_{2}^{*}\left(r\right)+i2\pi f\left(r\right)={𝓇}^{+}\left|\chi^{+}\left(r\right)\right|+{𝓇}^{-}\left|\chi^{-}\left(r\right)\right|+i\gamma \cdot d*\left(\chi^{+}\left(r\right)+\chi^{-}\left(r\right)\right)$$

Here, $\chi^{+}\left(r\right)$ and $\chi^{-}\left(r\right)$ are volumetric susceptibilities of positive and negative sources, ${𝓇}^{+}$ and ${𝓇}^{-}$ are their corresponding relaxometry constants [22], $d\left(r\right)=\left(2z^{2}-x^{2}-y^{2}\right)/4\pi {\left|r\right|}^{5}\;$is the dipole kernel, f(r) is the local field, and $R_{2}^{*}\left(r\right)$ is the transversal signal decay rate. The inverse problem of Equation (1) can be formulated as a minimization problem and solved iteratively using a conjugate gradient descent algorithm with Gauss–Newton iterations [24]. In the present work, the following formulation was implemented [19,22,25]:

$$\chi^{{+}^{*}},\chi^{{-}^{*}}=\underset{\chi^{+},\chi^{-}}{\mathrm{argmin}}||w_{1}{\left(R_{2}^{*}-\left({𝓇}^{+}\chi^{+}-{𝓇}^{-}\chi^{-}\right)\right)||}_{2}^{2}+||w_{2}{\left(f-d*\left(\chi^{+}+\chi^{-}\right)\right)||}_{2}^{2}+R\left(\chi \right)$$

where

(2) $$R\left(\chi \right)=2\lambda_{1}{\left|M_{mag}\nabla \left(\chi^{+}+\chi^{-}\right)\right|}_{1}+\lambda_{1}{\left|M_{r}\nabla \chi^{+}\right|}_{1}+\lambda_{1}{\left|M_{r}\nabla \chi^{-}\right|}_{1}$$

Here, ${𝓇}^{+}$ and ${𝓇}^{-}$ are relaxometric constants equal to 274 Hz/ppm [22], $\lambda_{1}$ is the regularization parameter, ∇ is a gradient operator, $M_{mag}$ is a binary edge mask derived from the magnitude image [25], $M_{r}$ is a binary edge mask derived from $R_{2}^{*}$ similar to $M_{mag}$. The data weight $w_{2}$ reflects the reliability of the estimated frequency of each voxel [25,26], while $w_{1}$ reduces the effects of unreliable $R_{2}^{*}$ estimations. Susceptibility values violating physical constraints ($\chi^{+}>0$ and $\chi^{-}<0$) were reset to zero. $\chi^{+}\left(r\right)$ and $\chi^{-}\left(r\right)$ were initialized to the solution of the system of equations at each voxel:

$$\{\begin{array}{c} R_{2}^{*}={𝓇}^{+}\left|\chi^{+}\right|+{𝓇}^{-}\left|\chi^{-}\right|\\ \chi =\chi^{+}+\chi^{-} \end{array}$$

where χ is the conventional QSM value. Regularization parameter $\lambda_{1}$ was set to 1500 in all reconstructions

The solver was implemented in Matlab (Mathworks, Natick, MA, USA).

### 2.2. Postmortem Tissue Imaging

The Institutional Review Board determined that activities involved in the present study did not constitute human subjects research as the project did not involve identifiable private information from or about living subjects. As a result, neither IRB approval nor a notice of exemption was required.

Data acquired in four formalin-fixed postmortem brain slabs were analyzed. Formalin-fixed postmortem brain specimens were embedded in 1% agarose gel to minimize motion and provide MR-visible medium to measure the magnetic field generated by the tissue. The tissue blocks were not washed prior to embedding. The tissue was scanned on a 3T clinical MRI scanner (GE Healthcare, Waukesha, WI, United States) using an 8-channel head coil. A three-dimensional multi-echo gradient echo (GRE) sequence with unipolar readout gradient was acquired for susceptibility mapping and $R_{2}^{*}$ with the following parameters: voxel size = 0.5 × 0.5 × 0.5 mm^3^, first TE = 5.9 ms, ΔTE = 5.9 ms, #TE = 8, TR = 52.2 ms, flip angle = 12 degrees, receiver bandwidth (rBW) = 244 Hz/pixel, acquisition time 69 min. For lesion identification, T2 FSE (voxel size = 0.5 × 0.5 × 2 mm^3^, TE = 60.3 ms, TR = 6332 ms, flip angle = 111°, rBW = 195 Hz/pixel, number of excitations (NEX) = 12, acquisition time 36 min) was acquired.

### 2.3. Data Processing

Prior to source separation, $R_{2}^{*}$ was estimated using Auto-Regression on Linear Operations (ARLO) [27]. To estimate the frequency maps, the multi-echo phase data was fitted to a nonlinear model [26]. Then, the result was spatially unwrapped [28], after which a background field was removed using Projection onto Dipole Fields (PDF) [29]. QSM was reconstructed using Morphology Enabled Dipole Inversion (MEDI) [25]. Co-registration of acquired $T_{2}w$ images was performed using the FSL FLIRT algorithm [30,31]. Each lesion of interest was subdivided into core and rim ROIs based on QSM reconstructions. Additionally, neighboring normal appearing white matter (NAWM) was segmented using ITK-SNAP (version 3.8.0; http://itksnap.org/, accessed on 10 June 2022). Tracings were reviewed by an experienced neuroradiologist, and average $\chi^{+}$ and $\chi^{-}$ measurements (referenced to the surrounding agarose) were recorded for each ROI.

All processing was performed on a desktop PC (CPU: Intel i7-5820k, 3.3 GHz; 64 GB RAM).

### 2.4. Immunohistochemistry

Lesions of interest were excised, embedded in paraffin, and cut into 5 μm sections. Sections were deparaffinized in xylene, rehydrated, and antigen retrieval was performed with 10 mM sodium citrate buffer (pH 6) for 20 min. Sections were quenched, blocked, and incubated overnight with a primary antibody against myelin basic protein (MBP, Dako A0623, 1:500), CD68 (microglia/macrophages; CellSignaling #76437, 1:500), followed by the appropriate biotinylated secondary antibodies and avidin/biotin staining kit with diaminobenzidine (DAB) as the chromogen (Vector Laboratories ABC Elite Kit and DAB Kit). Negative controls included isotype-controls and the absence of immunolabeling in tissues that do not express MBP or CD68. DAB-enhanced Perls’ Prussian blue was used to detect ferric iron. Slides were immersed in 4% ferrocyanide/4% hydrochloric acid for 30 min in the dark, and staining was enhanced through incubation with DAB for 30 min at room temperature. After staining, all sections were rinsed, dehydrated, cover-slipped, and digitized using a Mirax digital slide scanner.

### 2.5. Histology Optical Density Estimation

Regions of interest (3.341 ± 0.002 mm^2^) were manually drawn within the lesion and adjacent normal appearing white matter by a reader with 5 years of experience (KG). NAWM ROIs were sampled 0.57 ± 0.24 mm from the outer rim edge in iron− lesions and 1.24 ± 0.52 mm from the outer rim edge in iron+ lesions. For each lesion, we placed 1 ROI in the NAWM, 1 ROI in the rim (lesion perimeter for rim-negative lesions), and 1 ROI in the center. Lesions were defined histologically by the absence of MBP staining in the center. Histology ROIs were captured in Panoramic Viewer (version 1.15.4). Each image was processed in FIJI [16] where color deconvolution was applied to generate three images: hematoxylin, DAB, and residual (grayscale range [0…255]). Mean grey values from the DAB channel were averaged and used to calculate optical density (OD) using the following equation [17]: $OD=\log_{10}\left(\frac{255}{Mean\;Grey\;Value}\right)$.

### 2.6. Statistical Analysis

Statistical analysis and linear regressions between the ODs and magnetic susceptibilities were performed using Matlab toolboxes. Differences in susceptibilities and OD measured in lesion ROIs were investigated for significance using Wilcoxon signed rank test. In all statistical tests, the level of significance was chosen to be 0.05.

## 3. Results

### 3.1. Tissue Composition

**Figure 1:**
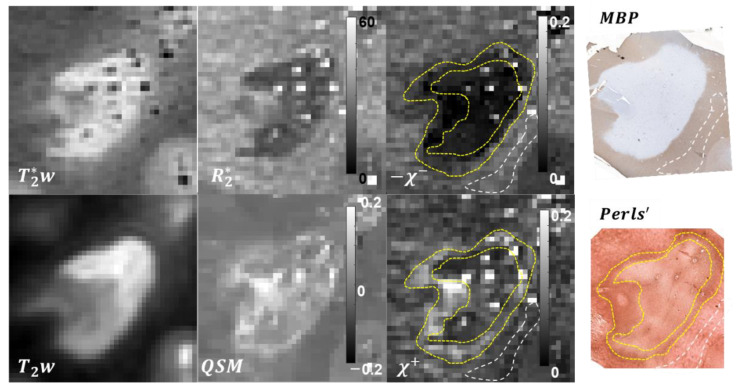
Results of the $R_{2}^{*}$-based separation of magnetic sources in a chronic active lesion. Paramagnetic lesion rim readily identifiable in QSM and $\chi^{+}$ (yellow dashed line) appears to be in good morphological agreement with the iron distribution revealed by Perls’ staining. Similarly, strong demyelination of the lesion core estimated with the proposed method is well reflected by the MBP staining. NAWM is shown with white dashed line.

**Figure 2:**
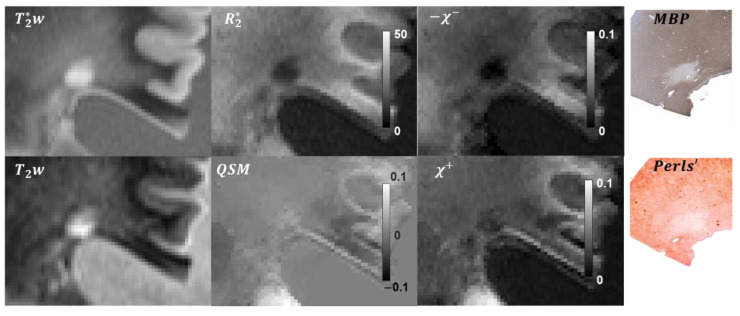
Example of the $R_{2}^{*}$-based separation of magnetic sources in a chronic silent lesion. The lesion appears to be weakly paramagnetic in the susceptibility map, with the Perls’ and MBP staining suggesting almost complete loss of myelin and partial loss of iron within the lesion ROI. These findings were similarly reflected in the estimated $\chi^{+}$ and $\chi^{-}$ maps.

In total, 10 lesions were included in the analysis. Based on CD68 staining, seven of the lesions were classified as chronic active, one as chronic silent, and two as actively demyelinating. Representative $\chi^{+}$ and $\chi^{-}$ maps and corresponding QSM, $R_{2}^{*}$, $T_{2}w$**,** and magnitude images and histological stains of MS lesions are shown in Figure 1 and Figure 2. Typical reconstruction $\chi^{+/-}$ reconstruction time was 15 min.

For the chronic active lesion (Figure 1), QSM depicted the lesion core almost isointense compared to NAWM and a pronounced paramagnetic rim; corresponding MBP staining showed almost uniform depletion of myelin, while Perls’ demonstrated iron rim with heterogeneous distribution of iron in the lesion core. These visual findings were in good qualitative correspondence with distribution of $\chi^{+}$ and $\chi^{-}$ sources estimated with proposed $R_{2}^{*}QSM$ method.

A chronic silent lesion (Figure 2) appearing weakly hyperintense on QSM was characterized by strong depletion of myelin according to the MBP staining and a minor decrease of iron concentration. Estimated distributions of $\chi^{+}$ and $\chi^{-}$ had a similar appearance, indicating greater depletion of paramagnetic sources compared to diamagnetic.

**Figure 3:**
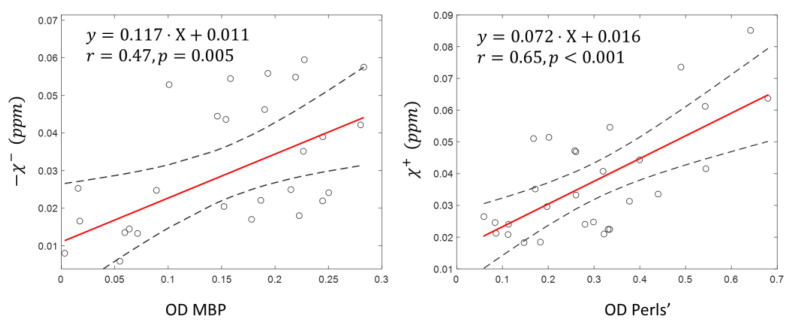
Correlation analysis between the average lesion/NAWM ROI source susceptibility and corresponding optical density of the histological stains.

Linear regression analysis between estimation of the optical density of the MBP/Perls’ histology and mean susceptibility of the corresponding sources within the ROIs of lesion and NAWM demonstrated a statistically significant correlation (MBP/$\chi^{-}$: correlation coefficient r = 0.47, *p* < 0.01, Perls’/$\chi^{+}$: r = 0.65, *p* < 0.001) (Figure 3).

### 3.2. Lesion ROIs

In the comparison of $\chi^{+}$ values and Perls’ OD obtained in lesion rim ROIs against the lesion core and NAWM, the positive susceptibility component (mean 0.052 ppm, 95%CI [0.036…0.068] ppm) and Perls’ OD (mean 0.376, 95%CI [0.235…0.517]) in lesion rim showed a statistically significant increase compared to lesion core ($\chi^{+}$: mean 0.034 ppm, 95%CI [0.026…0.042] ppm; OD: mean 0.228, 95%CI [0.15…0.306]) and normal-appearing white matter ($\chi^{+}$: mean 0.03 ppm, 95%CI [0.022…0.038] ppm; OD: mean 0.290, 95%CI [0.182…0.398]), demonstrating a similar trend in both modalities. Similarly, there were significant depletions of negative susceptibility component in lesion core ($-\chi^{-}$: mean 0.01 ppm, 95%CI [0.005…0.014] ppm; OD: mean 0.055, 95%CI [0.025…0.086]) compared to NAWM ($-\chi^{-}$: mean 0.051 ppm, 95%CI [0.046…0.056] ppm; OD: mean 0.192, 95%CI [0.153…0.230]), paralleled by decrease in MBP OD.

## 4. Discussion

Our data demonstrate the validity of the recently proposed $R_{2}^{*}QSM$ approach to separate colocalized positive and negative susceptibility sources by combining signal magnitude decay modeling and phase-based QSM reconstruction. The histological results obtained in a set of ex vivo MS lesions show statistically significant correlation with optical density of myelin- and iron-specific histological stains.

The main idea in the $R_{2}^{*}QSM$ framework is the proportionality between susceptibility and $R_{2}^{*}$ decay rate. This can be viewed as a reasonable assumption, as the gradient echo magnitude decay rate $R_{2}^{*}$ is dominated by static dephasing of susceptibility sources that is linearly dependent on concentrations of susceptibility sources and transverse relaxation enhancement by susceptibility sources is small [23,32,33,34,35,36,37]. The relaxometry constant close to the theoretic value 321 Hz/ppm [23] used in this study and our prior in vivo study seems to work well, and further investigation is needed to clarify uncertainty regarding relaxometry constants in literature [19,20]. There may be a constant component unaccounted in Equation (1) [17]; however, this constant term is likely small as indicated by the small $R_{2}^{*}$ value of ventricular cerebrospinal fluid that can be regarded as having no susceptibility source [38,39]. This $R_{2}^{*}$ model of susceptibility combined with QSM modeling of local field in Equation (2) results in successful susceptibility source separation without additional data acquisition such as needed for $R_{1}$ or $R_{2}$ mapping.

$R_{2}^{*}QSM$ estimation of iron and myelin would enhance the utilities of gradient echo MRI. While prior work has established clinical values of QSM for studying gray matter and MS lesion rim where paramagnetic iron dominates [40,41], QSM interpretation of white matter has been challenging. The $R_{2}^{*}QSM$ would improve the interpretation specificity for iron and myelin components. However, it should be noted that the $R_{2}^{*}$ modeling here and previous $R_{2}^{\prime }$ modeling of susceptibility ignore white matter anisotropy [42,43,44], which may contribute to the large spread in Figure 3 on the correlation between MBP OD and negative susceptibility. White matter myelin fiber orientations with respect to the main magnetic field in the brain can be estimated, for example, using diffusion tensor imaging. The data from this work and prior work on susceptibility source separation [16,17,19,21] suggests the feasibility of estimating an isotropic component of myelin’s susceptibility that seems approximately proportional to myelin concentration. Future development of susceptibility source separation should incorporate effects of microstructures, including myelin geometry for brain tissue, as well as precise background field inhomogeneity correction of $R_{2}^{*}$ to focus on tissue susceptibility source [45].

The optical density used in the present work is only semi-quantitative, which may also contribute to the large spread in the correlation between histology and susceptibility source separation in Figure 3. Nevertheless, this direct demonstration of agreement between histologic quantification and MRI-based $R_{2}^{*}QSM$ measurement of susceptibility components is encouraging. Future work should employ quantitative elemental analysis, such as simulated Raman scattering microscopy for specific quantitative mapping of myelin [46,47] and laser ablation inductively coupled plasma mass spectroscopy for iron quantitative mapping [7].

In conclusion, $R_{2}^{*}QSM$ separation of magnetic sources based solely on GRE complex data is feasible by combining $R_{2}^{*}$ magnitude decay rate modeling and QSM phase processing. This $R_{2}^{*}QSM$ quantification of para- and dia-magnetic sources simplifies acquisition protocols and allows broad applicability, including retrospective analysis of already existing data.

## Data Availability

The data presented in this study are available on reasonable request from the corresponding author.
